# Functionalized maghemite superparamagnetic iron oxide nanoparticles (γ-Fe_2_O_3_-SPIONs)-amylase enzyme hybrid in biofuel production

**DOI:** 10.1038/s41598-023-37826-2

**Published:** 2023-07-10

**Authors:** Samia S. Abouelkheir, Hassan A. H. Ibrahim, Ehab A. Beltagy

**Affiliations:** National Institute of Oceanography and Fisheries (NIOF), Alexandria, Egypt

**Keywords:** Biotechnology, Microbiology

## Abstract

The current study describes a straightforward, biologically and environmentally friendly method for creating magnetic iron oxide (γ-Fe_2_O_3_) nanoparticles. We report here that the *Bacillus subtilis* SE05 strain, isolated from offshore formation water near Zaafarana, the Red Sea, Hurghada, Egypt, can produce highly magnetic iron oxide nanoparticles of the maghemite type (γ-Fe_2_O_3_). To the best of our knowledge, the ability of this bacterium to reduce Fe_2_O_3_ has yet to be demonstrated. As a result, this study reports on the fabrication of enzyme-NPs and the biological immobilization of α-amylase on a solid support. The identified strain was deposited in GenBank with accession number MT422787. The bacterial cells used for the synthesis of magnetic nanoparticles produced about 15.2 g of dry weight, which is considered a high quantity compared to the previous studies. The XRD pattern revealed the crystalline cubic spinel structure of γ-Fe_2_O_3_. TEM micrographs showed the spherically shaped IONPs had an average size of 7.68 nm. Further, the importance of protein-SPION interaction and the successful synthesis of stabilized SPIONs in the amylase enzyme hybrid system are also mentioned. The system showed the applicability of these nanomaterials in biofuel production, which demonstrated significant production (54%) compared to the free amylase enzyme (22%). Thus, it is predicted that these nanoparticles can be used in energy fields.

## Introduction

Extensive research has been completed to explore the possible ways in which the enzymes can be exploited for biotechnological and environmental applications^1^. However, using free enzymes only shows some significant drawbacks, such as thermal instability, susceptibility to attack by proteases, activity inhibition, high sensitivity to numerous denaturing agents, and the impracticality of separating and reusing free catalysts at the end of the reaction^1^. A number of techniques are employed for avoiding these limitations and to improve the enzyme features and stability to be used under industrially relevant conditions.

The well-investigated procedure includes enzyme immobilization to improve storage of the enzymes^2,3^. Enzymes are immobilized on a solid support by surface-reactive functional groups^4^. Several nanoparticle-based supports, such as Au, Cu, silica, and others, have been utilized for the immobilization of different enzymes^5,6^. Immobilized enzymes have the advantage of a wider pH range, improved thermal stability, increased enzyme activity, easy separation from the solution, and simple support regeneration, making them suitable as industrial catalysts^1^. Along with the above-discussed benefits, immobilization of the enzyme on magnetic nanoparticles (MNPs) offers the extra benefits of biocompatibility, colloidal stability, superparamagnetic nature (high magnetization), easy magnetic separation, and recycling. The performance of an immobilized enzyme is governed by the structure and character of the carrier materials^7–10^. The use of MNPs in the industry provides a high surface area, which is advantageous for the high binding efficiency, increases the reaction rate, diminishes toxicity, lessens mass transfer resistance, and finally reduces fouling^9,11^.

One of the most important industrial processes is the hydrolysis of starch into low-molecular-weight sugars with higher economic values^12,13^. α-amylase or glycoside hydrolase hydrolyzes starch, glycogen, and relevant polysaccharides by cleaving the internal a-1, 4-glycosidic bonds in a random fashion^14^. These products are of considerable interest for industrial applications. α -amylase is in high demand in a variety of industries, including food, textiles, paper, the detergent industry, and medicinal applications^1^. Nowadays, the need for alternative fuel is a must as a consequence of the increasing price and depletion of crude oil and other non-renewable fossil fuels. Upon so doing, bio-ethanol was found to be the most common biofuel among the total biofuels, representing about 90%^15^. Seeking for an economically available substrate for bio-ethanol production, researchers found that starch was one of the best sources for biomass conversion through fermentation, where about 60% of bio-ethanol is produced^16^. In the light of such demand, crop residues plus biomass wastes were estimated to be the cheapest source for fermentation processes as renewable resources^17^. Furthermore, some countries established light gasoline, which began with 5% bioethanol in gasoline composition^18^.

There are very few studies dealing with the immobilization of enzymes on magnetic nanoparticles. Among these studies, Meridor and Gedanken describe an attempt to fabricate water-soluble-α-amylase nanoparticles (NPs) in a one-step sonochemical process^1^. The high thermal stability of this enzyme makes it suitable for NPs preparation. A solid surface was used to immobilize the newly formed NPs enzyme with a spherical morphology. Dhavale and his co-workers optimized the immobilization of α-amylase on chitosan-coated Fe_3_O_4_ MNPs (AMNPs) by varying enzyme concentration and incubation time^4^. Furthermore, the immobilized α-amylase was tested for starch hydrolysis under various physicochemical conditions, and its activity was compared to that of the free enzyme.

While Ajinkya and his team reported that γ-Fe_2_O_3_ nanoparticles have gained technological importance due to their magnetic and catalytic properties^19^, iron oxide nanoparticles (IONPs) with a small size of about 10–20 nm have a wide range of significant applications in electronics^20^, biomedicine^21^, energy^22,23^. The ability of the *B. subtilis* bacterium to reduce Fe_2_O_3_ is yet to be demonstrated. Therefore, the present study, aimed at developing a biological synthetic route for the cost-effective, non-toxic production of iron oxide nanoparticles through the use of a newly isolated *B. subtilis*, is believed to be highly promising. Having efficient, unconventional, and time-safe recycling tools for maximum starch fermentation in terms of nanomagnetic amylase in order to obtain bioethanol was also investigated.

## Results and discussion

**Figure 1:**
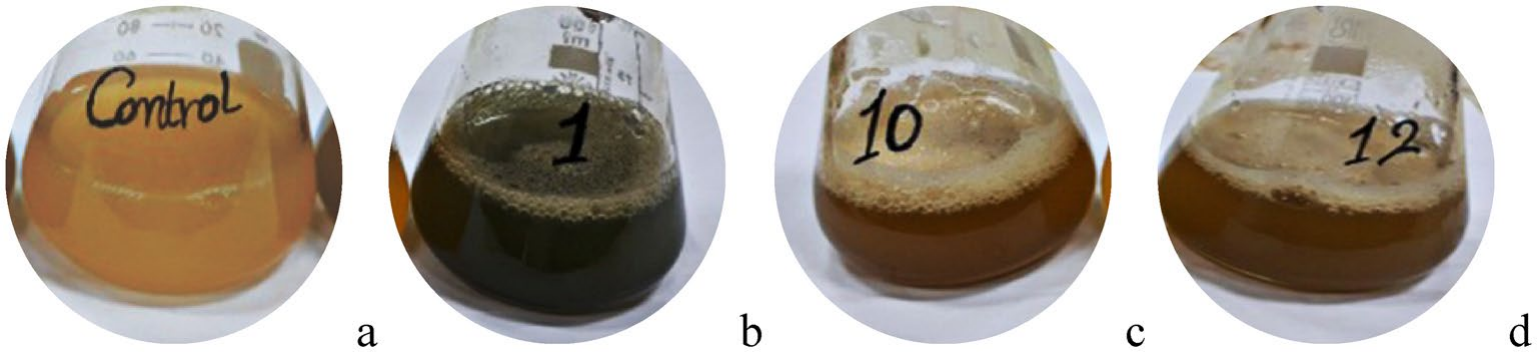
Shows the screening of the bacterial isolates (1, 10, and 12) that produce MIONPs following the colour conversion in comparison to the ferrous sulphate heptahydrate salt solution used as the control (FeSO_4_.7H_2_O).

Magnetic iron oxide nanoparticles (MIONPs) synthesis by isolated bacteria was evaluated visually by the colour change to the brown or black solution. Positive results found among three isolates out of thirteen recovered from different sources sediments (El-Hamra Lake, Wadi El-Natrun; Zafarana-offshore, Red Sea; Gabal El-Zeit-off shore, Red Sea; Ras Gemsha reef, Red Sea; North Geisum island reef, Red Sea, Hurghada) and formation waters (Gabal El-Zeit-off shore, Red Sea; Zafarana-offshore, Red Sea) (Fig. 1).

**Figure 2:**
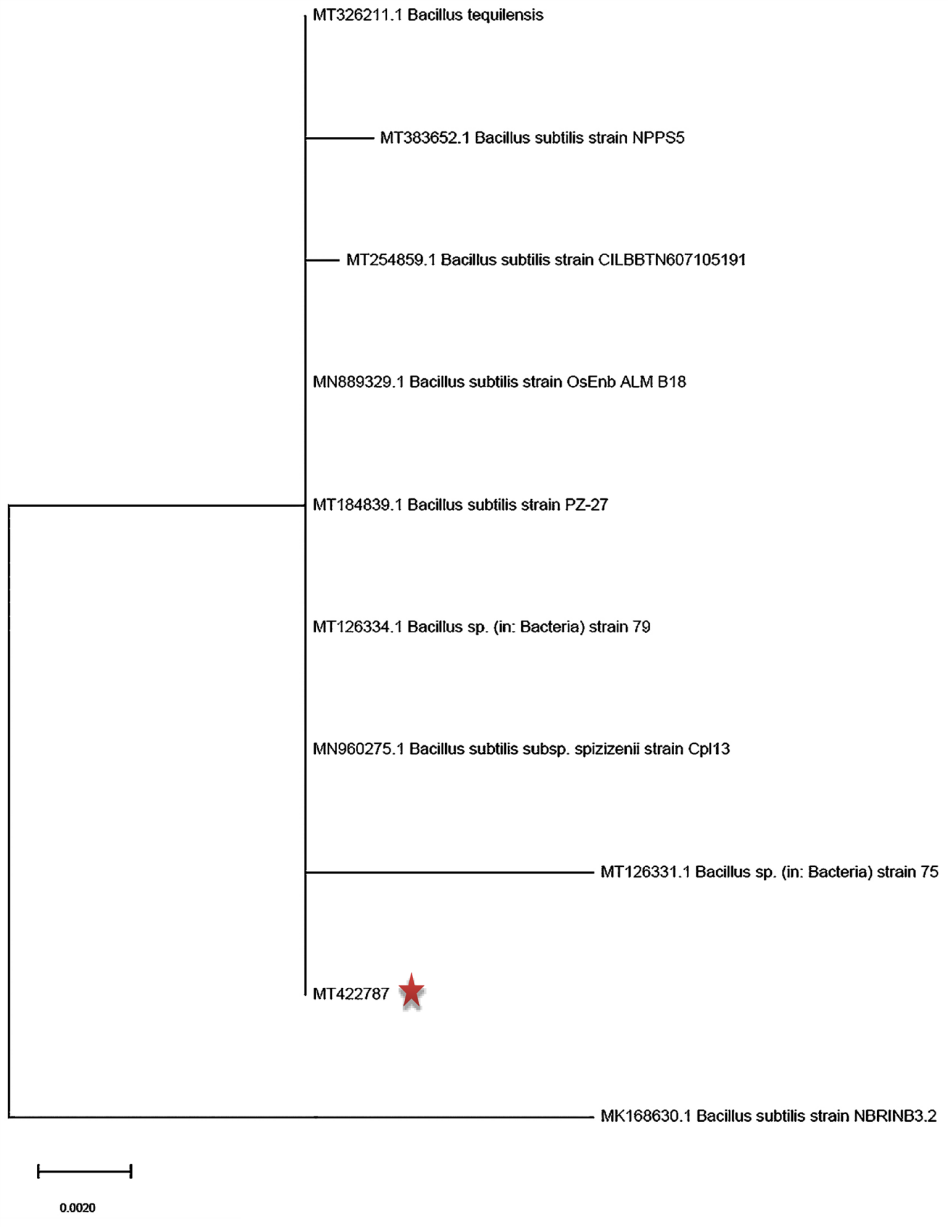
A phylogenetic tree based on the 16SrRNA sequence of *Bacillus subtilis* SE05 and the closely related bacterial species.

A promising isolate that synthesized magnetic iron oxide nanoparticles (MIONPs) through colour change to a dark solution was characterized as Gram + ve rod-shaped. The isolated bacterium from formation water (Zafarana-offshore, Red Sea) was identified as *Bacillus subtilis* SE05 based on 16S rRNA sequence that showed 100% similarity to *Bacillus subtilis* strain NPPS5 (accession number MT383652.1) and *Bacillus subtilis* strain PZ-27 (MT184839.1). Accordingly, the sequence was deposited in the National Center for Biotechnology Information (NCBI) GeneBank under the accession number MT422787 (https://www.ncbi.nlm.nih.gov/nuccore/MT422787). The phylogenetic tree of the 16S rRNA of *Bacillus subtilis* SE05 with its relation to the available sequences on the NCBI GenBank database illustrated in Fig. 2. This tree is important tool for organizing knowledge of biological diversity. It contains a lot of information about the inferred evolutionary relationships and communicates hypothesized evolutionary relationships among nested groups of taxa (monophyletic groups) that are supported by shared traits known as synapomorphies. The phylogenetic tree analysis can serve as the foundation for "evolutionary synthetic biology," which helps us better understand the evolution of cellular pathways, macromolecular machines, and other emergent properties of early life.

**Figure 3:**
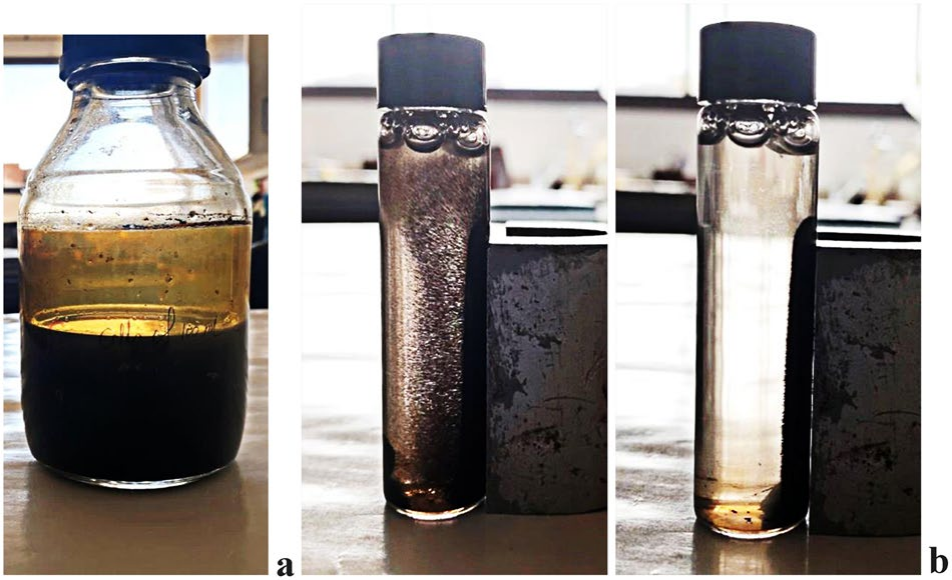
γ-Fe_2_O_3_-SPIONs synthesis using *Bacillus subtilis* SE05 cells (**a**), and nanoparticles purification using an external magnet (**b**).

The identified strain *Bacillus subtilis* SE05 was cultured in nutrient broth (NB) at pH 7 and 30 °C under shaken growth conditions 24 h. Around one gram bacterial cells were used to synthesize magnetic iron oxide nanoparticles (MIONPs) that appeared as a black powder dispersed in solution (Fig. 3). The MIONPs produced were purified using an external magnet. It was noticed that after 24 h of incubation, *Bacillus subtilis* SE05 synthesized an average of 15.2 g/L dried black powder.

*Bacillus subtilis* has been reported previously for silver and gold nanoparticle synthesis by both extracellular and intracellular means. Moreover, *Bacillus subtilis* strains isolated from rhizosphere soil succeeded in IONPs production^24^. This successful synthesis of stabilized Fe_3_O_4_ NPs capped by organic molecules allows the bulk synthesis of IONPs by the isolated *Bacillus subtilis* strain. Also, Wu and his colleagues^25^ reported that the bacterium *Actinobacter* sp. was capable of synthesizing γ-Fe_2_O_3_NPs with the help of Bharde and his co-workers^26^ under aerobic conditions when reacted with a ferric chloride precursor. However, the ability of *Bacillus subtilis* bacterium to reduce Fe_2_O_3_ has yet to be demonstrated. The expected mechanism used by the bacterium to reduce Fe_2_O_3_ and the end product of this reduction may be as follows: According to the current paradigm, dissimilatory iron-reducing bacteria (DIRB) and sulfate-reducing bacteria (SRB) activity results in the accumulation of a variety of reactive iron oxides, hydroxides, and sulphides as respiratory end products.

Therefore the present work described for the first time the capability of this strain at developing a biological synthetic route for the cost-effective production of γ-Fe_2_O_3_ iron oxide nanoparticles that are believed to be highly promising.

### X-ray diffraction analysis of functionalized γ-Fe_2_O_3_ nanoparticles

**Figure 4:**
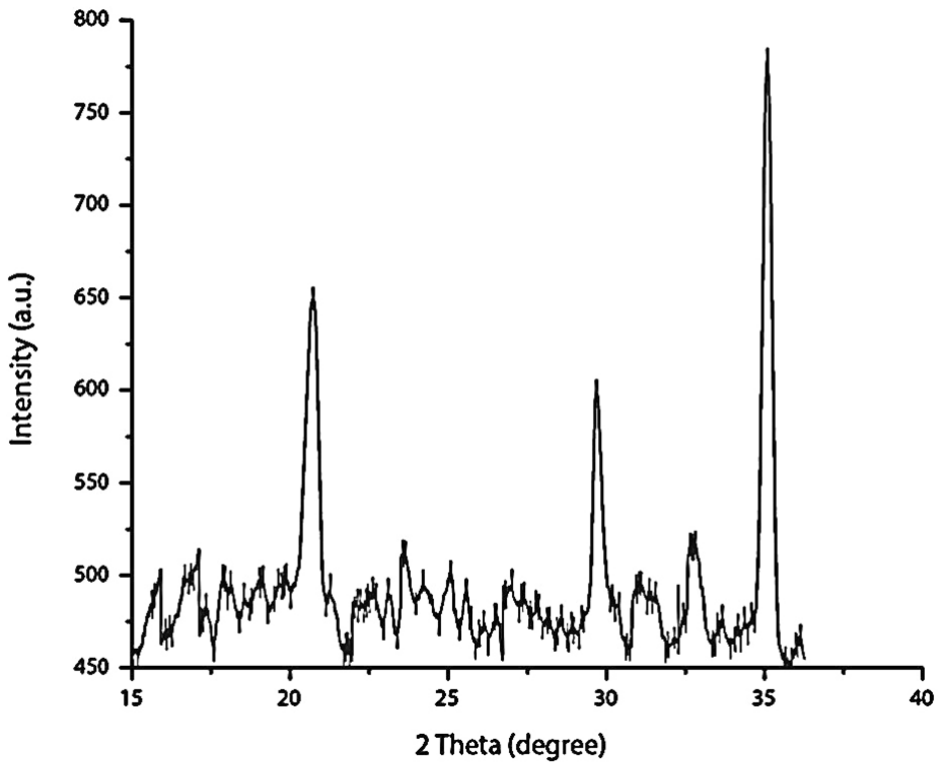
XRD pattern of iron oxide γ-Fe_2_O_3_ nanoparticles.

The structural and crystalline nature of the synthesized MIONPs was examined using X-ray diffraction analysis. The peak position and relative intensity of the reflection peaks of the XRD pattern of Fe_2_O_3_ (Fig. 4) confirm the crystalline cubic spinel structure of the γ-Fe_2_O_3_. The average grain size of the MIONPs nanoparticles calculated from the characteristic peaks at 21.409°, 29.720°, and 35.043° was ∼17 nm. These peaks for γ-Fe_2_O_3_ are corresponding to the lattice planes (hkl) < 210 >, < 211 >, and < 311 >, respectively. XRD data obtained in this study is well-matched with that reported by Ruíz-Baltazar and his co-workers^27^ and Guivar and his colleagues^28^ who confirmed the presence of the main diffraction peak in all patterns associated to γ-Fe_2_O_3_ phase is at 2θ = 35.5°. There are two additional peaks located at 23.77° (210) and 26.10° (211) in the XRD pattern associated with the γ-Fe_2_O_3_ phase. These intensities can be used to differentiate between the magnetite and maghemite phases^29,30^.

### FTIR analysis of functionalized γ-Fe_2_O_3_ nanoparticles

**Figure 5:**
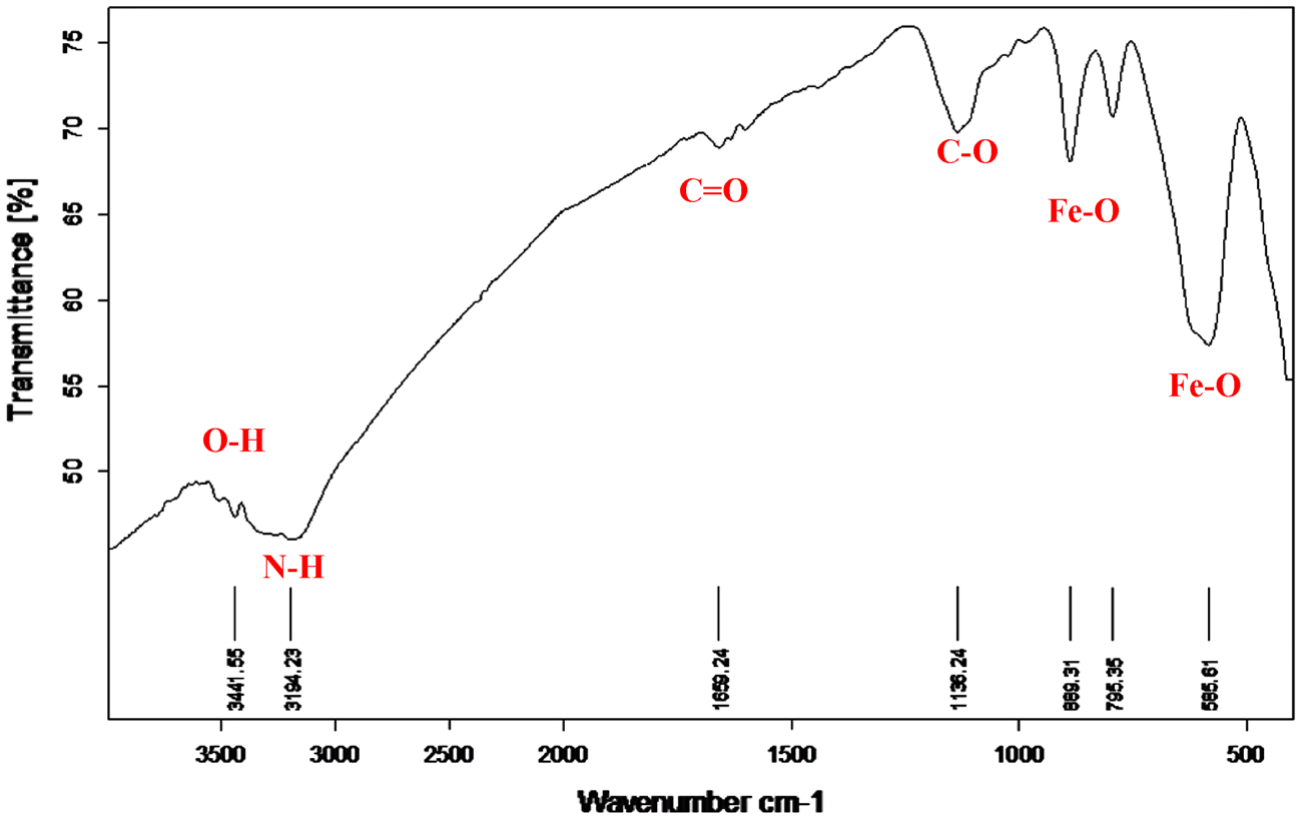
FT-IR spectrum of γ-Fe_2_O_3_ nanoparticles.

The FT-IR spectrum of biosynthesized γ-Fe_2_O_3_ nanoparticles demonstrated a strong absorption band at 585.6145 cm^−1^ due to the stretching vibration of Fe–O bonds between 850 and 400 cm^−1^. The peaks around 3441.5520 and 3194.2283 cm^−1^ appeared in the spectrum correspond to the O–H group where bands between 3200 and ~ 3600 cm^−1^ assigned for the organic functional group of hydroxyl (O–H) or –NH groups of phenols, alcohols, or carboxylic acids. One peak appeared at 1659.2419 cm^−1^ for the carbonyl group (C=O); peaks between 1730 and 1625 cm^−1^ match C=O stretch due to the capping of carboxylic acid. Finally, one peak at 1136.2414 cm^−1^ assigned to C–O stretching of the primary alcoholic group while the presence of C–O Stretch in between 1300 and 1000 cm^−1^ may be due to the covalent linking of ester or ether groups to the nanoparticle. Taking that into consideration, these functional groups on the surface functioned as surface coating and stabilizing agents. The net results of the FT-IR analysis (Fig. 5) in this study matches well the previously mentioned by Sundaram and his colleagues^24^ there were certain organic compounds that contain O–H, C=O, C–O, N–H groups act as a capping agent in the biosynthesized γ-Fe_2_O_3_ nanoparticles.

### Morphology and size determination of functionalized γ-Fe_2_O_3_ nanoparticles using TEM

**Figure 6:**
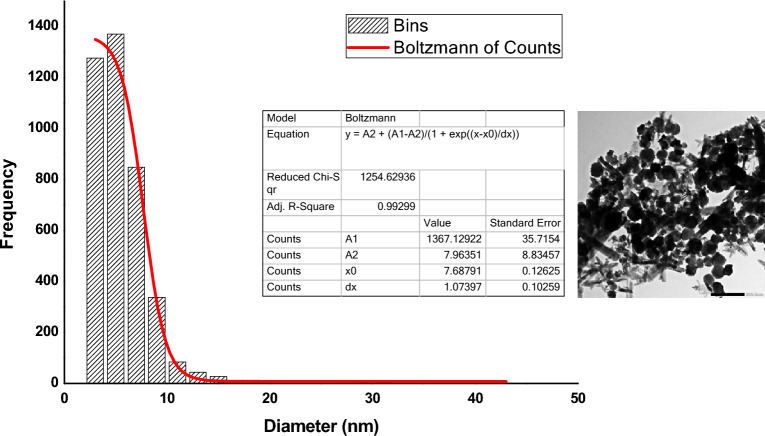
TEM image of γ-Fe_2_O_3_ nanoparticles shows the spherical shape of the synthesized particles and size distribution histogram with an average size of 7.68 nm.

Further analysis of the produced γ-Fe_2_O_3_ nanoparticles was carried out by Transmission Electron Microscope (TEM). The image described the morphology of the nano-sized γ-Fe_2_O_3_ particles that showed a spherical shape nature with an average size of 7.68 nm determined from the size distribution histogram (Fig. 6).

The difference in the crystallite size calculated from X-ray diffraction about 17 nm and the size obtained from TEM of 7.68 nm is because of the several crystallization domains of particles detected by X-rays while the whole particle with TEM. In good agreement with the results obtained, Bharde and his co-workers previously succeeded in synthesizing two types of iron-based magnetic nanoparticles using the bacterium *Actinobacter* sp.: maghemite (γ-Fe_2_O_3_) and greigite (Fe_3_S_4_) under ambient conditions, depending on the nature of the precursor's used^26^. The bacterium synthesized γ-Fe_2_O_3_ when it reacted with ferric chloride and iron sulfide when exposed to the aqueous solution of ferric chloride-ferrous sulfate, and the average particle size was found to be 19 nm^26^.

### Magnetic studies of functionalized γ-Fe_2_O_3_ nanoparticles using VSM

**Figure 7:**
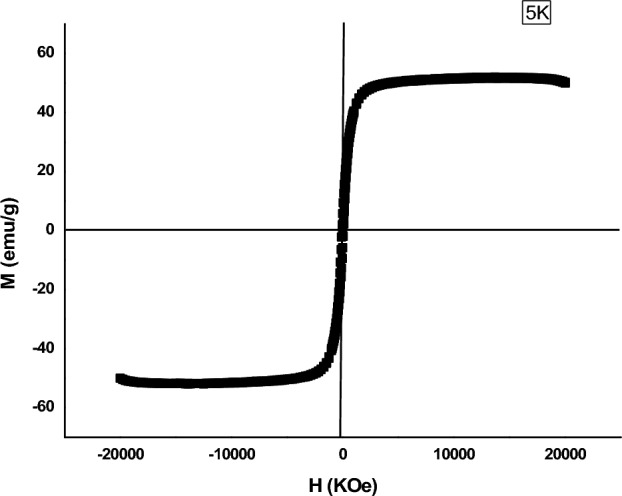
Magnetization curve of γ-Fe_2_O_3_ nanoparticles.

The γ-Fe_2_O_3_ particles can exhibit unusual magnetic behaviors at the nanometer size scale that are reasonably different from those of bulk ones. These magnetic properties were dependent on the morphology and crystal structure of the sample^31^. The magnetic properties of the synthesized γ-Fe_2_O_3_ nanoparticles were studied using VSM (Fig. 7). The magnetic hysteresis loops of γ-Fe_2_O_3_ nanoparticles in the applied magnetic field sweeping from − 20,000 to 20,000 KOe showed much-closed loops with 51.989 emu/g saturation magnetization (Ms). These closed hysteresis loops resulted in small coercivity (Hci) (74.628 Oe) and retentivity (Mr) (5.8005 emu/g) providing good quality of the superparamagnetic property. Moreover, γ-Fe_2_O_3_ NPs showed superparamagnetic characteristics as expected and previously mentioned by Wu and his colleagues^25^.

The small hysteresis loop and low coercivity observed from the analysis of the γ-Fe_2_O_3_ nanoparticles confirming the small particle size as mentioned previously by Rafi and his co-workers^31^.

### Purity study on functionalized γ-Fe_2_O_3_ nanoparticles using EDX analysis

**Figure 8:**
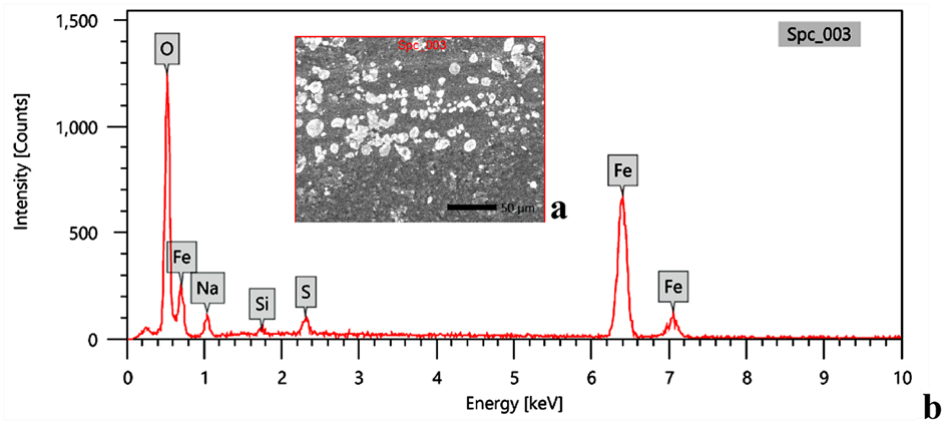
EDX analysis of synthesized γ-Fe_2_O_3_ nanoparticles; (**a**) Selected area, (**b**) EDX spectra.

The purity of the synthesized γ-Fe_2_O_3_ nanoparticles was examined by Energy Dispersive X-ray (EDX) analysis. EDX spectral analysis appeared in Fig. 8 reveals the presence of sodium, silicon, sulfur, oxygen, and iron in the synthesized γ-Fe_2_O_3_ nanoparticles with mass % 5.37 ± 0.28, 0.71 ± 0.08, 1.93 ± 0.10, 36.63 ± 0.40, and 55.36 ± 0.74, respectively. The purity of the metal oxide (FeO) produced was 91.99%. The appearance of these peaks in EDX spectra is due to the functional groups of the capping agents through the biological synthesis.

### Zero and in-field Mössbauer spectroscopy of functionalized γ-Fe_2_O_3_ nanoparticles

**Figure 9:**
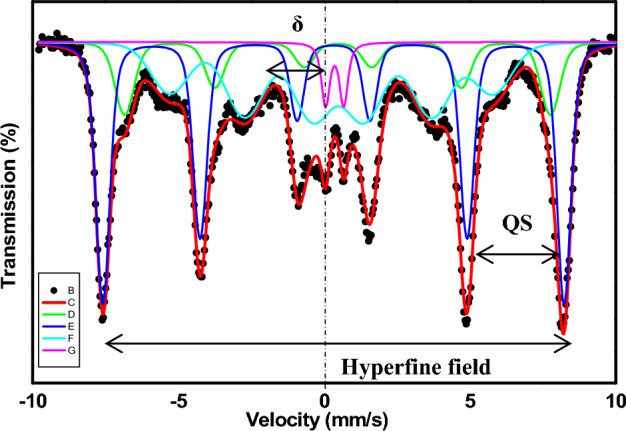
Mössbauer spectrum of ^57^Fe in functionalized γ-Fe_2_O_3_ nanoparticles.

Maghemite and magnetite nanoparticles cannot be distinguished from one another by X-ray powder diffraction; hence, Mössbauer spectroscopy was used to do so. Both experimental and analytical spectra are shown in Fig. 9 and Table 1.

**Table 1:** The Mössbauer Hyperfine parameters of ^57^Fe in functionalized γ-Fe_2_O_3_ nanoparticles.

γ-Fe_2_O_3_	δ (mm/s)	QS (mm/s)	HWHM (mm/s)	Hhf (KOe)
Double line absorption peak (trivalent) 1	0.359	0.623	0.23	
Six-line absorption peak 2	0.466	−0.023	0.23	452.820
Six-line absorption peak 3	0.319	−0.005	0.23	490.520
Six-line absorption peak 4	0.367	−0.241	0.25	343.480

It can be seen from Fig. 9 that a six-line spectrum and sub-spectra appear in the Mössbauer field, demonstrating that the sample is magnetic. In good accordance with the results obtained and the previously published Mössbauer Spectroscopic Analysis^32^ that found the isomer shift (δ = 0.40 mm/s), quadrupole splitting (QS = 0.72 mm/s), line width (0.49), and hyperfine field (497.2 KOe) of the standard Mössbauer spectrum of γ-Fe_2_O_3_ samples for octahedral coordination, B site, and 0.2 mm/s, 0.59 mm/s, 0.44, and 497.3 KOe for tetrahedral coordination, A site, respectively. To the best of our knowledge, the isomer shift (δ) movement of Fe^3+^ is commonly around 0.40 mm/s; therefore, according to the Mössbauer parameters in Table 1, we can define that the valence of iron in all samples is + 3. The γ-Fe_2_O_3_ sample presented in Fig. 9 has four sets of Mössbauer parameters at 300 K and typically comprises two types of iron atoms in tetrahedral and octahedral configurations. Subspectrum 4 with a regular energy shift (δ) of 0.367 mm/s may result from iron atoms located in an octahedron inside the crystal lattice. Subspectrum 3 with a regular energy shift (δ) of 0.319 mm/s may originate from iron atoms occupying tetrahedral positions in the crystal lattice. Subspectrum 2 with a regular energy shift (δ) of 0.466 mm/s may be derived from iron atoms on the surface of the lattice. These parameters are close to those of the bulk γ-Fe_2_O_3_ sample. While the double-line peaks in the spectrum 1 (δ = 0.359) may be attributed to the sample containing a limited number of extremely small-sized nanoparticles with superparamagnetic properties.

### Functionalized γ-Fe_2_O_3_-amylase enzyme hybrid system preparation

**Figure 10:**
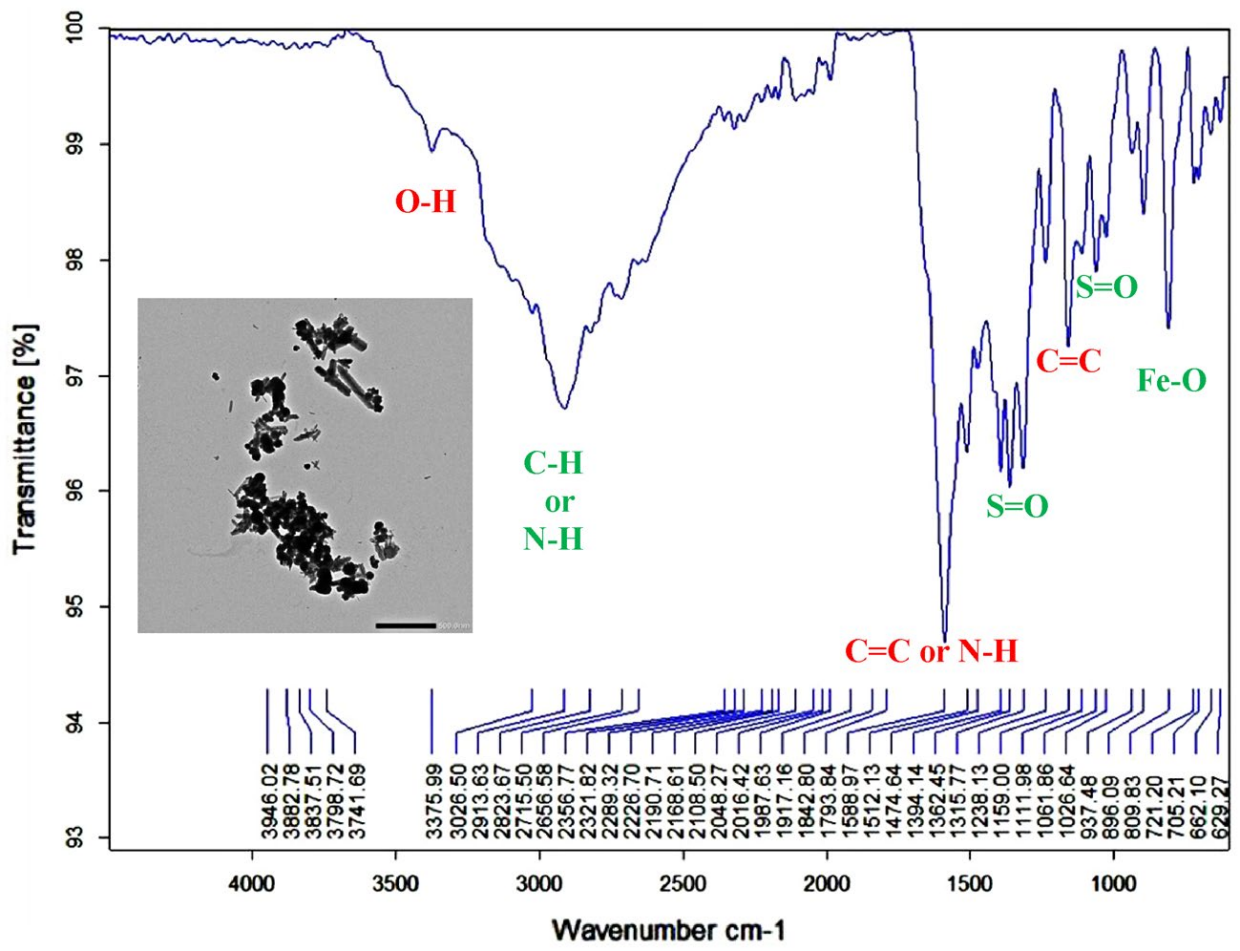
FTIR analysis of synthesized γ-Fe_2_O_3_ -amylase enzyme hybrid system.

γ-Fe_2_O_3_-amylase enzyme hybrid system prepared using the characterized synthesized γ-Fe_2_O_3_ nanoparticles and amylase enzyme. The fabrication of the hybrid system was confirmed using TEM and FTIR analyses (Fig. 10).

The FTIR pattern of the immobilized amylase enzyme onto a γ-Fe_2_O_3_ surface was examined and compared to the FTIR pattern of the γ-Fe_2_O_3_ nanoparticles only (Table 2). The hybrid pattern consolidated the existence of new functional groups not appeared in FTIR of the nanoparticles at a wavenumber of 2913.63, 1315.77, 1061.86, and finally 705.21 cm^−1^ those corresponding to the C–H or N–H, S=O, S=O, and Fe–O functional groups not found in the FTIR pattern of nanoparticles only. Taking into consideration that the biological synthesis of magnetic nanoparticles allows many functional groups on the surface that functioned as a surface coating, shape controllers, and stabilizing agents which confirmed by the FTIR analysis performed during the study for magnetic nanoparticles (O–H, N–H, C=O, C–O, Fe–O) only (Fig. 5) and synthesized γ-Fe_2_O_3_ -amylase enzyme hybrid system (O–H, N–H or C-H, C=C or N–H, S=O, C=C, Fe–O) (Fig. 10).

**Table 2:** FTIR spectra and the peak positions of the major IR bands of γ-Fe_2_O_3_ nanoparticles only and γ-Fe_2_O_3_-amylase enzyme hybrid.

Standard peak wavenumber range cm^−1^	Peak wavenumber of γ-Fe_2_O_3_ cm^−1^	Peak wavenumber of γ-Fe_2_O_3_-amylase enzyme hybrid cm^−1^	Bond and compound class	Standard peak reference
3200–3600 cm^-1^	**3441.5520**	**3375.99**	**O–H stretching, strong, alcohol**	^1,4,29^
2500–3300 cm^-1^	**3194.2283**	**3026.50**	**O–H stretching, carboxylic acid**	^29^
2500–3300 cm^-1^		**2913.63**	**O–H stretching (carboxylic acid), N–H stretching (amine salt), C-H stretching (alkane)**	^1,28^
		2823.67	Symmetric stretching of –CH_2_	^1,8,28^
		2356.77	P–H group, atmospheric CO_2_	^24,29^
		2321.82	P–H group	^24^
		2289.32	P–H group	^24^
		2226.70	P–H group	^24^
		1842.80	Alkyl, carboxyl and amino	^28^
		1793.84	C = O group of carboxylic acid	^1,24,28^
1650–1580 cm^-1^	**1659.2419**	**1588.97**	**N–H bending (amine), C = C stretching (cyclic alkene)**	^4^
		1512.13	ν asCOO − , δ-NH_2_, C = C, NH^3+^	^28^
		1474.64	–CH_2_ rocking band	^1^
		1394.14	COO − symmetric stretching	^10^
		1362.45	COO − symmetric stretching	^10^
1350–1300 cm^-1^		**1315.77**	**S = O, Strong stretching (Sulfone)**	^8^
		1238.13	C–O stretching (carboxylic acid)	^1^
1124–1205 cm^-1^	**1136.2414**	**1159.00**	**C = C bending, strong, alkene**	^28^
		1111.98	Si–O–Si asymmetric stretching	^8,28^
1070–1030 cm^-1^		**1061.86**	**S = O stretching, strong (sulfoxide)**	^8^
		1026.64	Iron oxyhydroxide (γ-FeOOH)	^26^
		937.48	Silanol groups	^8^
885–895 cm^-1^	**889.3135**	**896.09**	**C = C bending, strong, alkene**	^28^
790–840 cm^-1^	**795.3530**	**809.83**	**C = C bending, medium, alkene**	^28^
		721.20	–CH_2_ rocking band	^1^
500–800 cm^-1^		**705.21**	**Fe–O stretching, strong**	^28^
		**662.10**	Fe–O bond vibration	^26–28^
		**629.27**	Fe–O stretching vibration	^8^
500–600 cm^-1^	**585.6145**		**Fe–O stretching, strong**	^4,28^

Significant peak wavenumbers of γ-Fe_2_O_3_ only & γ-Fe_2_O_3_-amylase enzyme hybrid (cm^−1^) with their bonds & compound classes are in bold.

These function groups facilitate the bonding with the nanoparticles. This concept was confirmed by Guivar and his co-workers who stated that capping agents such as fatty acids form a protective monolayer, where carboxylate (R–COO −) groups are strongly bonded to the γ-Fe_2_O_3_ nanoparticles surface^28^.

### Reaction mixture yield course time

**Figure 11:**
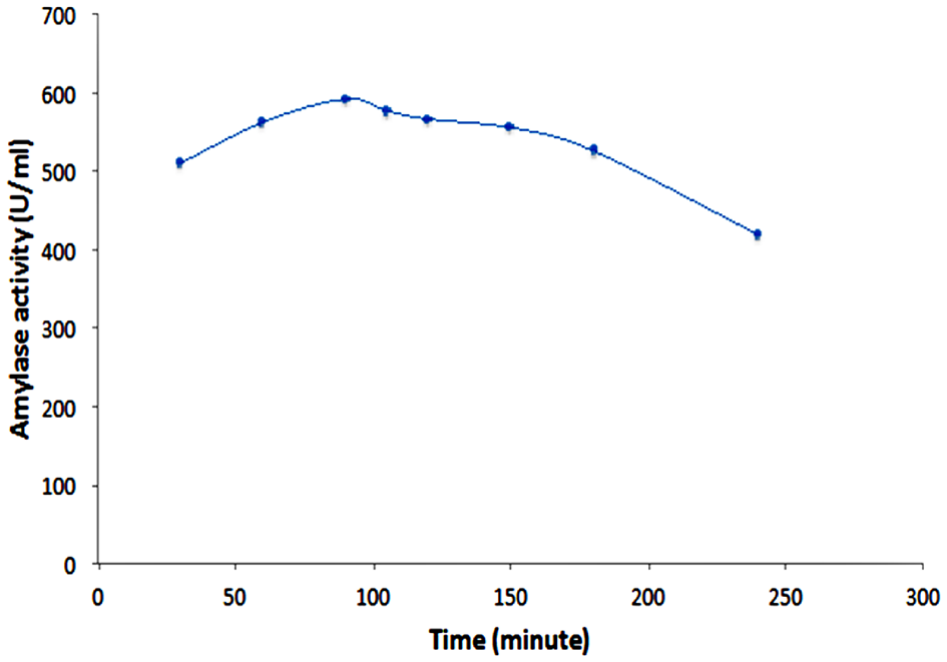
Time course of amylase isozyme activity.

Optimum incubation period for maximum amylase activity produced by *Bacillus subtilis* SE05 and administered as γ-Fe_2_O_3_-amylase enzyme hybrid achieved after 90 min incubation, the maximum amylase activity was 592.92 U/mg (Fig. 11).

### Cycling of functionalized γ-Fe_2_O_3_-amylase enzyme hybrid

**Figure 12:**
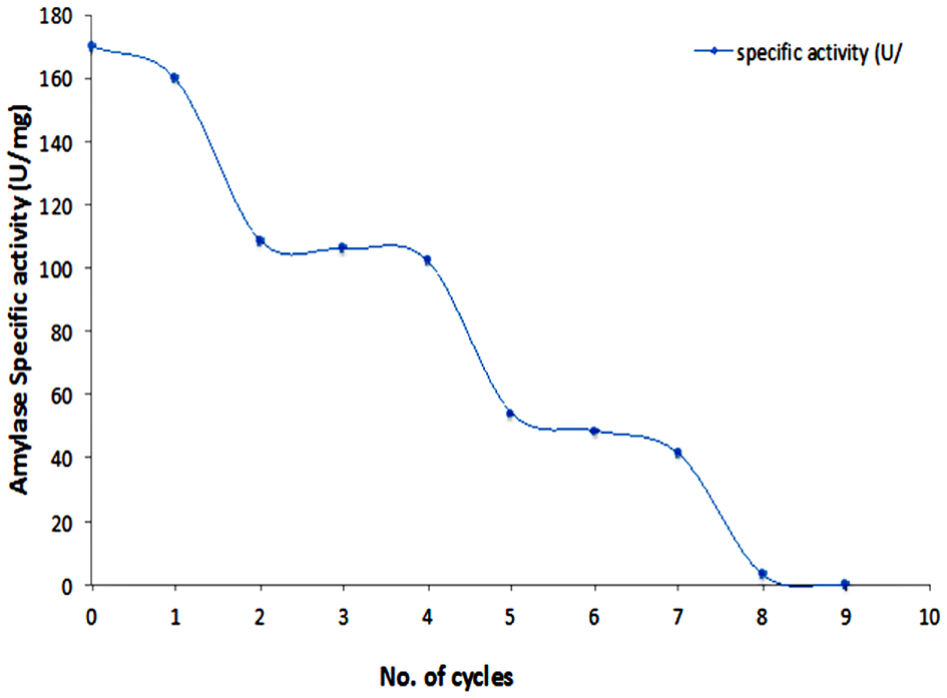
Cycling of γ-Fe_2_O_3_-amylase enzyme hybrid for 9 runs.

As demonstrated in Fig. 12, the nano-amylase was recycled for 9 cycles. Maximum specific activity was approximately 169.63 U/mg, followed by gradual decline pattern retaining about 50% of activity (76.73 U/mg) with cycle no. 5.

Likely, Baskar and his colleagues have found that α -amylase MNPs has kept about 65% of its initial activity till cycle no. 6th of potato starch hydrolysis at optimum condition^33^. However, Dhavale and his co-workers have reported the stability of amylase magnetic nanoparticles (AMNPs) activity till 5th cycle and potential recycling for 20 times keeping 79% of its initial activity^4^.

### Bioethanol production

Purified amylase isozymes of *Bacillus subtilis* SE05 in a shape of γ-Fe_2_O_3_-amylase enzyme hybrid directly administered to degrade glucose backbone presenting in starch in order to produce soluble sugar that could be used in bioethanol fermentation media. As indicated in Table 3, weighing starch (1.8 g/l) was enzymatically hydrolyzed by γ-Fe_2_O_3_-amylase hybrid giving 3.04 U/μg amylase in 90 min giving 10.4 g of soluble sugar. About 54% of such byproduct was converted by baker yeast (*Saccharomyces cerevisiae*) to bioethanol, representing about 2.89 (g/g/L), nevertheless, although the same weight of starch was hydrolyzed by free amylase giving 2.74 (U/μg amylase) and producing (9.4 g) soluble sugar, only 22% of those soluble sugars were converted by baker yeast to bioethanol (1.06 g/g/L).

**Table 3:** Bioethanol production by free and γ-Fe_2_O_3_-amylase hybrid.

	Free amylase (FA)	Nano-immobilized amylase (NIA) [NIA + Starch solution (24 h incubation) added to yeast (48 h incubation)]	NIA + Starch solution + Yeast (incubation 48 h)	Starch solution + Yeast (48 h incubation)
Enzyme activity (U/ug)	2.736	3.038	–	–
Sugar solution conc. (g/l)	9.4	10.4	–	–
Actual ethanol g/g/l	1.064	2.885	–	–
Theoritical ehtanol (g/l)	4.809	5.320	–	–
Conversion efficiency (%)	22	54	–	–
Ethanol conc. value by Dichromate assay (%)	1	3	0.3	0.05

Such results implied that the conversion efficiency of the γ-Fe_2_O_3_ -amylase hybrid was twice that of the free ones in the case of sequential fermentation, where amylase byproducts were added to yeast cultures for fermentation in a separate step. Notably, non-significant results were obtained from direct incubation of starch with yeast (*Saccharomyces cerviceae*) only for 48 h as well as incubation of starch with nano-immobilized amylase and yeast as a batch for 48 h in terms of simultaneous fermentation.

The most likely explanation of such a case is that free-adding soluble sugar to yeast culture without any nano-material could exert an osmotic pressure on yeast cells, providing a stress condition that creates what is called feedback inhibition for glucoamylase^34,35^. On the other hand, adding nanomagnetic amylase combined with soluble sugars to yeast cultures might help reduce the pressure on yeast cells by aiding the yeast cells metabolize and convert the sugars to bioethanol more efficiently in a sense of a quick fermentation process, in other words, nourishing the yeast cells growth^34^.

The current study indicated that sequential fermentation (Seq. F) gave the highest conversion rate (52%), compared to simultaneous fermentation (SF). Such a finding contradicts the finding of Larrea and his team, who reported that SF is much better than Seq. F for high bioethanol yield^18^. Shanavas and his co-workers have found that the maximum ethanol yield was 0.11 g/g^36^, which is lower than the maximum ethanol yield in the current study (2.89 g/g/L), yet is a lower value than other reported ones^35,36^. Bearing in mind that 3.04 U per μg of immobilized amylase-specific activity has provided such value, adding more immobilized amylase isozymes to starch would raise the yield of bioethanol. Vesna and his colleagues have supported that, who mentioned that adding more amylases to high starch content would result in a higher ethanol yield^37^.

### Qualitative measurement of bio-ethanol by GC –Mass analysis

**Figure 13:**
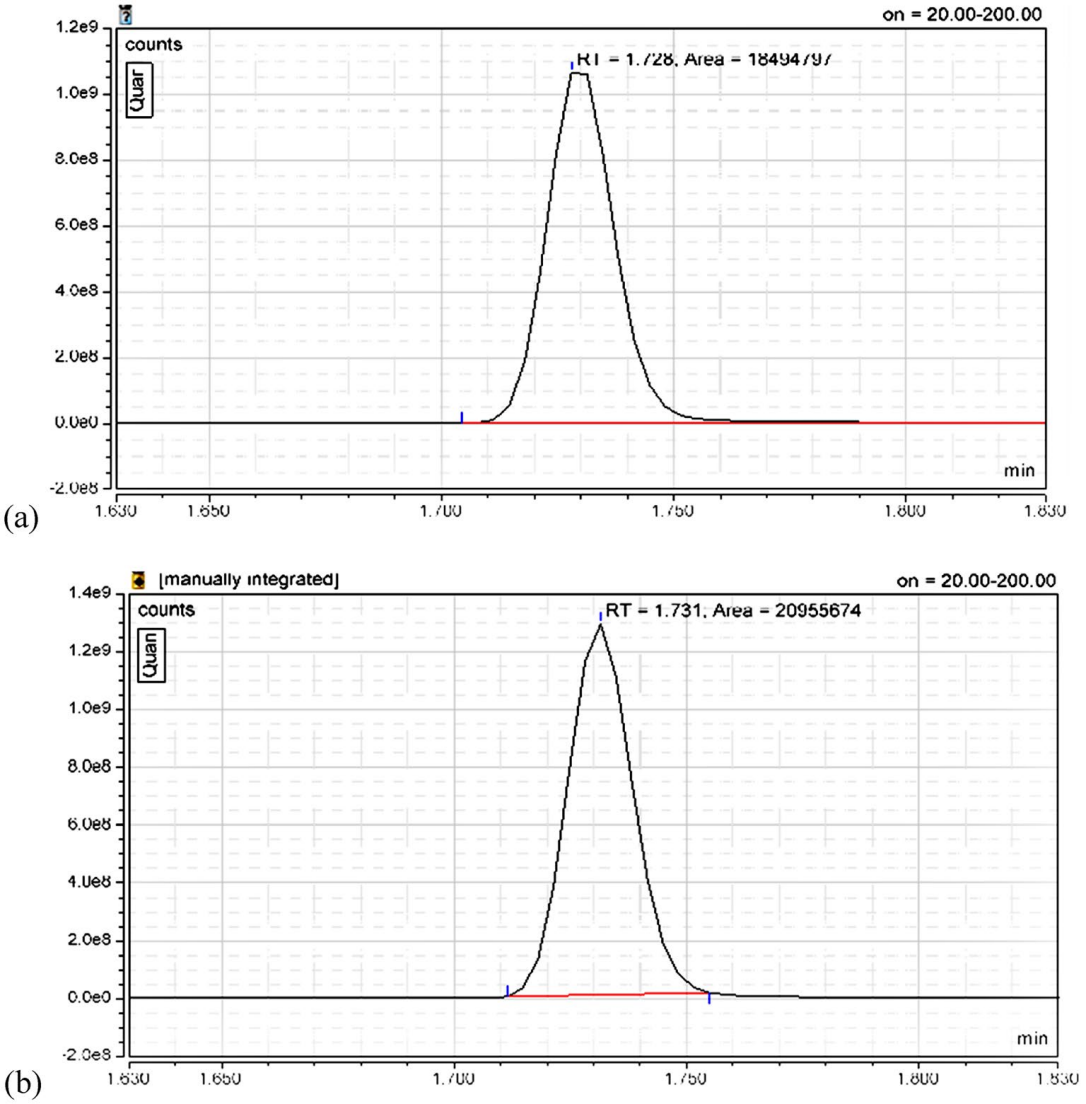
GC-Mass chromatogram: (**a**) ethanol product detection at retention time 1.728 min produced by γ-Fe_2_O_3_ -amylase hybrid activity and fermentation of degraded starch; (**b**) ethanol standard peak at retention time 1.731 min.

The bioethanol product was confirmed qualitatively using GC-Mass by matching the ethanol peak resulted from fermented sugar solution using nano-amylase hybrid plus commercial yeast (*Saccharomyces cerviceae*) at RT 1.728 min as shown in Fig. 13a and the given control one at RT 1.731 min (Fig. 13b).

## Conclusion

Discovering novel magnetic nanoparticles in terms of their unique characteristics and features is a matter of interest. Furthermore, the high potential for producing bio-ethanol as a biofuel from starch using immobilized amylase as a γ-Fe_2_O_3_—amylase hybrid with highly specific activity and a recyclable option provides a cost-effective and environmentally friendly way to manage starch-rich wastes.

## Materials and methods

### Isolation sites

Mesophilic iron-reducing bacteria were isolated from different growth niches such as sediment and water collected from El-Hamra Lake, Wadi El-Natrun; Zafarana offshore, Red Sea; Gabal El-Zeit offshore, Red Sea; Ras Gemsha reef, Red Sea; North Geisum island reef, Red Sea, Hurghada sediments and Gabal El-Zeit offshore, Red Sea; Zafarana offshore, Red Sea formation waters.

### Bacteriological media

Medium composition is given in gL^−1^ unless otherwise stated. The medium was prepared with seawater and sterilized by autoclaving at 121 °C for 20 min. Isolation was performed on YSG agar medium, which contained yeast extract (2), glucose (1), soluble starch (2), and agar (20); the pH was adjusted to 3 with 1 M H_2_SO_4_^38^. Nutrient agar medium (Oxoid) was used for screening and seed culture preparation and was composed of peptone (5); beef extract (3); and NaCl, 5. Before solidifying with 20 g agar the pH was adjusted to 7 with NaOH^39^. Bioethanol production medium consisting of (g/l): peptone, 20; yeast extract, 10; and pH 5.5.

### Bacterial isolation

Each sample (1 mL) was dispensed in flasks containing 100 mL of YSG agar medium broth and incubated at 30ºC. The isolation from sediment samples was performed using the serial dilution technique^40^. One milliliter from each flask was used to inoculate agar plates. Plates were incubated at 30 degrees Celsius. Colonies were picked up and purified using the classical purification techniques.

### Iron substrate

Salt solutions (1 mM) of ferrous sulphate heptahydrate (FeSO_4_.7H_2_O) (Nice Chemicals, Egypt) and ferric chloride anhydrous, 98% extra pure (FeCl_3_) (LOBA Chemie, India) were purchased of analytical grade and prepared using deionized water.

### Screening for MIONPs producers

The ability of pure culture isolates to synthesize iron oxide nanoparticles was investigated by a grown-up in a 250-mL Erlenmeyer flask containing 100 mL of nutrient broth medium with continuous shaking on a rotary shaker (150 rpm) at 30 °C for 24 h. Salt solutions of FeCl_3_ and FeSO_4_.7H_2_O were prepared (10^−3^ M) and mixed with the whole culture. The entire mixture was kept on a rotary shaker at 30 °C and150 rpm. The reaction was permitted to continue for a period of 24 h. The biotransformed solution was observed by the colour transformation to black.

### Bacterial identification

A partial 16S rDNA fragment of the proposed strain was amplified using universal primers. The forward primer (F27) was AGAGTTTGATCMTGGCTCAG, and the reverse primer (R1429) was CGGTTACCTTGTTACGACTT, while the standard PCR protocol was used in the following conditions: denaturation at 95 °C for 5 min, followed by 30 cycles of 3 segments, including denaturation at 95 °C for 30 s, annealing at 65 °C for 30 s and elongation at 72 °C for 1 min, the end of the program was the final extension at 72 °C for 7 min. The resultant PCR product was sent to LGC Company in Germany for sequencing^41^. The bacterial strain used in this study was maintained in glycerol culture at −80 °C. For experimentation purposes, an aliquot of glycerol culture was cultivated on Nutrient Broth (NB) medium (LAB M, United Kingdom) and maintained at 37 °C.

### MIONPs biosynthesis

Magnetic iron oxide nanoparticles (MIONPs) were prepared based on biological synthesis. In a typical synthesis, 50 mM of ferrous sulphate (FeSO_4_·7H_2_O) substrate was inoculated with 1 g of cells of the identified isolate. The mixture was incubated and kept at 37 °C under shaken conditions at 150 rpm. Incubation time continued for 24 h to allow the growth of the NPs. The biotransformed solution was observed visually by the colour change to brown or black. An external magnet was used to separate the MIONPs produced. The MPs were washed three times with distilled water for further experiments.

### MIONPs characterization

The phase purity of the prepared samples was characterized by X-ray diffraction using a Bruker XRD-D2 Phaser (Bruker, Germany) X-ray diffractometer with Cu Kα radiations operated at a voltage of 30 kV and a current of 10 mA. The average particle size was estimated by using the Scherer formula: D = 0.9λ β cos θ Where λ is the X-ray wavelength (0.1541 nm), β is the FWHM (full width at half), θ is the diffraction angle, and D is the diameter size of the crystallite. The Mössbauer spectrum of CaFeO_3_-δ sample at room temperature was obtained using a conventional ^57^Fe constant acceleration spectrometer with ^57^Co (embedded in rhodium matrix) radioactive source. The Fourier transmission infrared (FTIR) spectra of the nanoparticles were recorded using a Fourier transmission infrared spectrometer (Bruker Tensor 37) in the range of 3500 to 500 cm^−1^. The morphology and size of the nanoparticles were studied using a transmission electron microscope (TEM). A Lakeshore 7410 vibrating sample magnetometer (VSM) was used to get the magnetic properties of the MIONPs. The purity of the synthesized nanoparticles was determined by energy dispersive X-ray (EDX) analysis using a scanning electron microscopy (SEM) (Jeol JSM-IT 200).

### MIONPs-amylase enzyme hybrid system preparation

The MIONPs -amylase enzyme hybrid system was prepared by mixing 100 mg of magnetic iron oxide nanoparticles (MIONPs) with 100 mL of amylase enzyme solution for 15 min at room temperature, then allowing it to settle overnight in the refrigerator. The hybrid system was extracted using an external magnet, washed, and dried. TEM and FTIR were used to characterize the newly formed hybrid system^42,43^.

### MIONPs application in biofuel production

#### Bioethanol production

Starch with ratio of 1.8% (w/v) was used for hydrolysis by amylase isozymes produced by *Bacillus* *subtilis* SE05 and semi-purified to their homogeneity by ammonium sulfate precipitation (data not shown) in both status (free and immobilized). The resulted byproduct in terms of soluble sugar was measured by DNS methods as described before, where those sugars were provided to bioethanol production medium^44^, in addition to inoculum size of 3 ml *Saccharomyces cerevisiae* culture. Then, the mixed culture was fermented at 30 °C for 48 h. Rotary evaporator was run at 50 °C seeking for evaporated ethanol from fermented medium. Dichromate assay as described by Yoswathana^45^ was applied for ethanol yield determination. Standard curve in range from 2 to 20% was plotted for slope deduction.

#### GC-mass analysis

The qualitative analysis of bioethanol was conducted by a GC-mass spectrometer (Thermo Scientific TSQ 9000–1 triple quadrupole GC–MS,) with the Chromeleon 7, Version 7.2.10, Thermo Fisher Scientific mass spectrometry software was worked according to procedure of Suleiman^46^. The standard curve was plotted with standard of different ethanol concentration from 10 to 30%. The concentration of ethanol was estimated with internal standard value of ethanol analysis by GC-Mass. The ethanol was identified with retention times (RT) and peak range.

### Statistical analysis

All experiments were performed in triplicates. Statistical evaluation of data as the mean ± SE was performed, plotted, and calculated using Origin Pro 8.1. The significant values were determined at *P*-value < 0.05.

## Data Availability

The datasets generated and analyzed during the current study are available from the corresponding author on reasonable request. The *Bacillus subtilis* SE05 sequence was deposited in the National Center for Biotechnology Information (NCBI) GeneBank under the Accession Number MT422787 (https://www.ncbi.nlm.nih.gov/nuccore/MT422787).
